# Efficacy of *Posidonia oceanica* Extract against Inflammatory Pain: In Vivo Studies in Mice

**DOI:** 10.3390/md19020048

**Published:** 2021-01-21

**Authors:** Laura Micheli, Marzia Vasarri, Emanuela Barletta, Elena Lucarini, Carla Ghelardini, Donatella Degl’Innocenti, Lorenzo Di Cesare Mannelli

**Affiliations:** 1 Department of Neuroscience, Psychology, Drug Research and Child Health (NEUROFARBA)‐Pharmacology and Toxicology Section, University of Florence, Viale Gaetano Pieraccini, 6, 50139 Florence, Italy; laura.micheli@unifi.it (L.M.); elena.lucarini@unifi.it (E.L.); carla.ghelardini@unifi.it (C.G.); 2 Department of Experimental and Clinical Biomedical Sciences, University of Florence, Viale Morgagni 50, 50134 Florence, Italy; marzia.vasarri@unifi.it (M.V.); emanuela.barletta@unifi.it (E.B.); donatella.deglinnocenti@unifi.it (D.D.); 3 Interuniversity Center of Marine Biology and Applied Ecology “G. Bacci” (CIBM), Viale N. Sauro 4, 57128 Livorno, Italy

**Keywords:** *P. oceanica*, inflammation, pain, CD-1 mice

## Abstract

*Posidonia oceanica* (L.) Delile is traditionally used for its beneficial properties. Recently, promising antioxidant and anti-inflammatory biological properties emerged through studying the in vitro activity of the ethanolic leaves extract (POE). The present study aims to investigate the anti-inflammatory and analgesic role of POE in mice. Inflammatory pain was modeled in CD-1 mice by the intraplantar injection of carrageenan, interleukin IL-1β and formalin. Pain threshold was measured by von Frey and paw pressure tests. Nociceptive pain was studied by the hot-plate test. POE (10–100 mg kg^−1^) was administered per os. The paw soft tissue of carrageenan-treated animals was analyzed to measure anti-inflammatory and antioxidant effects. POE exerted a dose-dependent, acute anti-inflammatory effect able to counteract carrageenan-induced pain and paw oedema. Similar anti-hyperalgesic and anti-allodynic results were obtained when inflammation was induced by IL-1β. In the formalin test, the pre-treatment with POE significantly reduced the nocifensive behavior. Moreover, POE was able to evoke an analgesic effect in naïve animals. Ex vivo, POE reduced the myeloperoxidase activity as well as TNF-α and IL-1β levels; further antioxidant properties were highlighted as a reduction in NO concentration. POE is the candidate for a new valid strategy against inflammation and pain.

## 1. Introduction

*Posidonia oceanica* (L.) Delile is a marine vascular plant belonging to the Posidoniaceae family and the only endemic species of the Mediterranean Sea. It is a seagrass that blooms underwater forming vast meadows of tens of thousands of square kilometers of great ecological importance and is essential for the entire marine ecosystem [1]. 

According to tradition, *P. oceanica* provided benefits for human health. The first information on the *P. oceanica* healing properties comes from ancient Egypt, where it was assumed to be effective against sore throats and skin problems [2]. Other documents describe its traditional use to treat inflammation and irritation, but also acne, lower limbs pain and colitis [3]. 

A more recent tradition of the villagers of the west Anatolian coast concerns the use of *P. oceanica* leaves decoction as a natural remedy for diabetes and hypertension [4]. An in vivo preclinical study claimed that oral administration of an ethanolic extract from *P. oceanica* leaves in alloxan-induced diabetic rats lowered blood sugar, restored antioxidant enzyme activity and reduced the lipid peroxidation process, supporting the antidiabetic and vasoprotective roles of *P. oceanica* [4].

**Figure 1:**
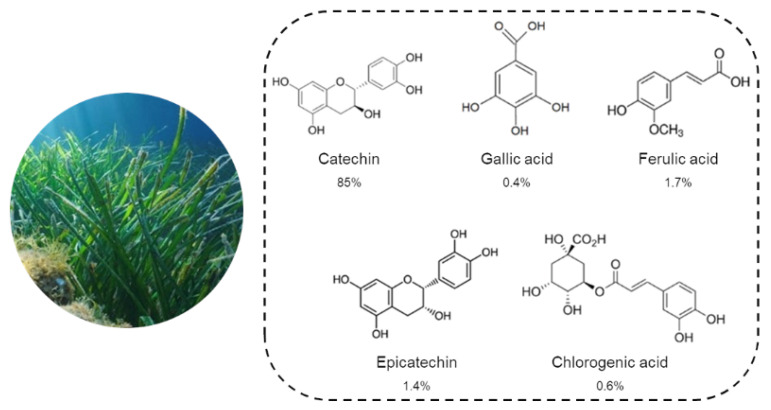
Phenolic profile of *P. oceanica* leaves extract (POE) obtained by UPLC analysis [5]. The percentage composition of each phenolic compound in POE is reported below each chemical structure. An additional 11% of POE composition remains unknown and/or uncharacterized.

Recently, the hydroalcoholic extract from *P. oceanica* leaves, called POE, has been the focus of a series of bioactivity studies. A first UPLC characterization analysis, conducted by some of our authors [5], showed that the hydrophilic fraction of POE consisted of 88% phenolic compounds. The polyphenolic profile was specifically represented by about 85% (+) catechins, while the remaining 5% by a mixture of gallic acid (0.4%), ferulic acid (1.7%), epicatechin (1.4%) and chlorogenic acid (0.6%). The small remaining fraction (11%) was represented by minor peaks, indicating the presence of further compounds, which, although detectable as phenols, are un-known/uncharacterized (Figure 1) [5].

Although the individual phenolic compounds identified have been tested in some experimental models of in vitro bioactivity [5], POE has been shown to be particularly effective as a phytocomplex. Indeed, POE has proved to be capable of inhibiting the migration of cancer cells, such as human fibrosarcoma HT1080 cells [5,6] and human neuroblastoma SH-SY5Y cells [7]. The total absence of cellular toxicity in POE activities has been attributed to its ability to modulate the activation of the autophagic process [6]. 

In relation to the traditional and recognized antidiabetic role of *P. oceanica*, POE has also proven to be an effective in vitro inhibitor of the protein glycation process, strengthening its potential use in the management of diabetic pathophysiology and associated complications [8]. 

In addition, some authors of this work have previously provided the first experimental support for the potential therapeutic application of POE against various inflammatory-associated disorders [9]. Indeed, POE was found to be able to effectively inhibit the LPS-induced inflammatory process in RAW264.7 murine macrophages, blocking the signaling cascades upstream of NF-κB, the crucial transcription factor for pro-inflammatory mediators’ production. 

Inflammation is a pathophysiological condition characteristic of many of the most life-threatening diseases in humans, encompassing pain as a main symptom. 

Conventional non-steroidal anti-inflammatory drugs (NSAIDs) are commonly prescribed to relieve pain and reduce inflammation. However, prolonged clinical use of NSAIDs is strongly discouraged due to their common, even serious, side effects [10]. Novel, safe, pharmacological approaches are necessary for treating, in particular, chronic inflammatory diseases. The use of herbal medicines is still today one effective strategy in the management of diseases and in relieving pain, as they are an important source of natural compounds with different bioactive properties [11]. 

The anti-inflammatory role of POE described above could be recognized as an innovative strategic weapon to fight the progression of these pathologies. Furthermore, the cell-safe POE profile [5,6,7,9] makes this phytocomplex an excellent candidate for the study of alternative natural strategies against inflammation in order to reduce the use of conventional drugs and, consequently, their side effects.

In light of these considerations, this work aims to investigate the effect of oral administration of POE on pain and inflammation in different models of acute inflammatory pain in CD-1 mice.

## 2. Results and Discussion

### 2.1. Biochemical Characterization and Antioxidant Activity of POE

The hydroalcoholic extraction method was able to recover polyphenols and carbohydrates from minced *P. oceanica* dried leaves.

Here, POE was found to contain 0.7 ± 0.02 mg/mL gallic acid equivalents of polyphenols and 10 ± 2.3 mg/mL glucose equivalents of carbohydrates. The antioxidant activity of POE was further evaluated by DPPH and FRAP assays. Particularly, POE exhibited radical scavenging and antioxidant activities of 1.2 ± 0.04 and 0.24 ± 0.05 mg/mL ascorbic acid equivalents, respectively.

The data were in agreement with those previously obtained [5].

### 2.2. The Effect of POE Against Inflammatory Pain

Inflammation is a physiological response to various stimuli (physical, chemical and biological or a combination) characterized by the recruitment and activation of immune cells, which rapidly manage the resolution and healing of damaged tissues [12].

Inflammation leads to the alteration of the pain threshold, inducing a pathological hypersensitivity, which represents the first passage from physiological nociception to persistent pain [13]. An uncontrolled immune response can make inflammation a pathological condition, so it is not surprising that inflammation and pain are key features of most human ailments.

In light of the recent discovery on the relevant in vitro anti-inflammatory effects of POE [9], the potential of POE to relieve pain in different models of acute inflammatory pain in vivo was investigated for the first time in this study.

Inflammatory pain was induced in mice by local injection of pro-inflammatory agents. The carrageenan model has been extensively used to study acute pain and inflammation [14,15]; in this work, carrageenan was intraplantarly administered to evoke a dramatic acute reaction characterized by pain and edema in mice.

**Figure 2:**
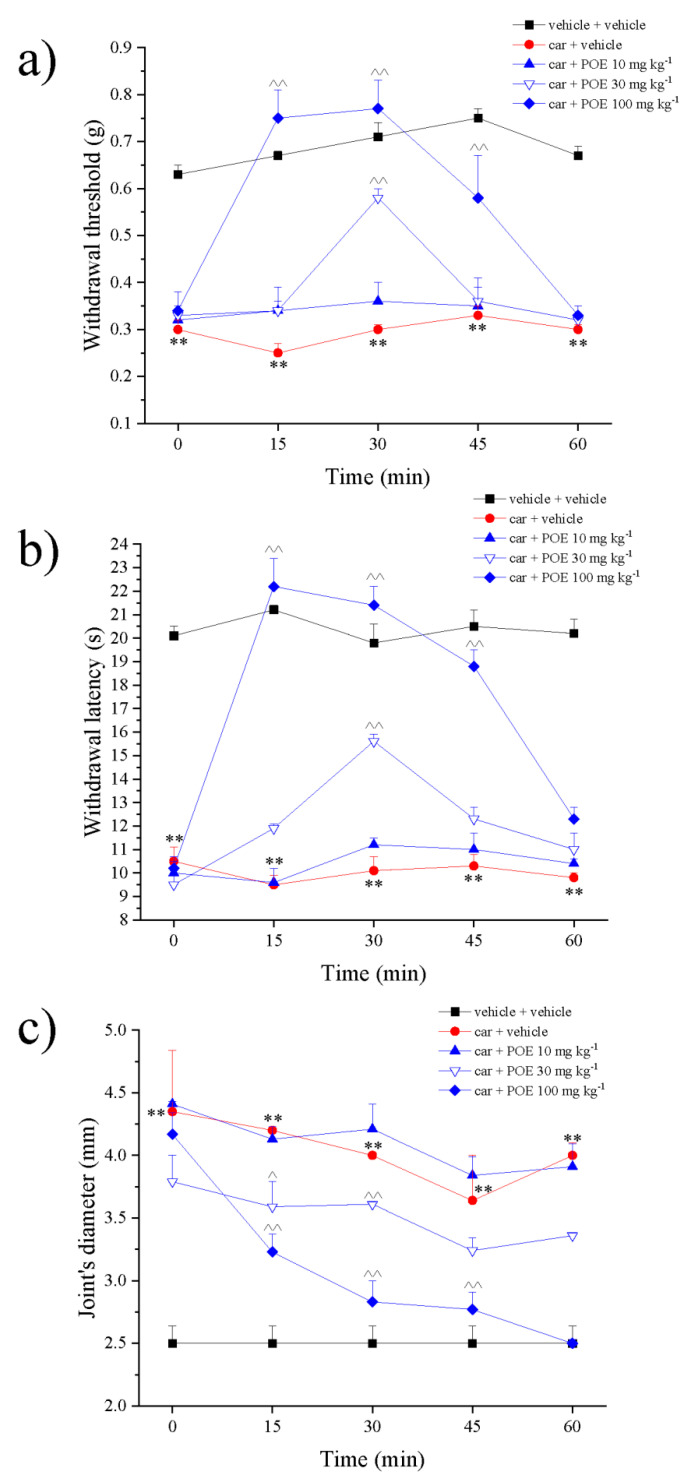
POE effects against carrageenan-induced pain and paw oedema. Two hours after the intraplantar injection of carrageenan (car), POE was per os administered. Pain threshold was measured by (**a**) von Frey test and (**b**) paw pressure test over time; (**c**) at the same time points, oedema was evaluated by measuring the joint’s diameter. Results are reported as mean ± S.E.M. of 10 mice analyzed in 2 different experimental sessions. ** *p* < 0.01 vs. vehicle + vehicle; ^^ *p* < 0.01 vs. car + vehicle.

In Figure 2a, pain threshold measurement by von Frey test is reported. Non-noxious mechanical paw stimulation (allodynia-like measure) allowed us to observe a decreased withdrawal response in carrageenan-treated animals that maintained a plateau between 2 and 3 h after treatment. Administration of POE (10–100 mg kg^−1^) in a dose-dependent manner increased the pain threshold; the higher dose was significantly effective between 15 and 45 min after treatment, completely blocking carrageenan-induced hypersensitivity.

POE efficacy was confirmed by paw pressure test, the extract was able to counteract carrageenan-dependent pain even when evoked by a noxious mechanical stimulus (hyperalgesia-like response), as illustrated in Figure 2b. POE 30 and 100 mg kg^−1^ also reduced the joint’s diameter made edematous by carrageenan (Figure 2c); POE 100 mg kg^−1^ was fully effective even 60 min after administration.

The carrageenan-induced acute and local inflammation consists of two phases. The early phase (0–1 h) is related to the production of histamine, serotonin and bradykinin, as first mediators, while the second phase has been linked to the production of prostaglandins and various cytokines such as IL-1β, IL-6, IL-10 and TNF-α [16].

**Figure 3:**
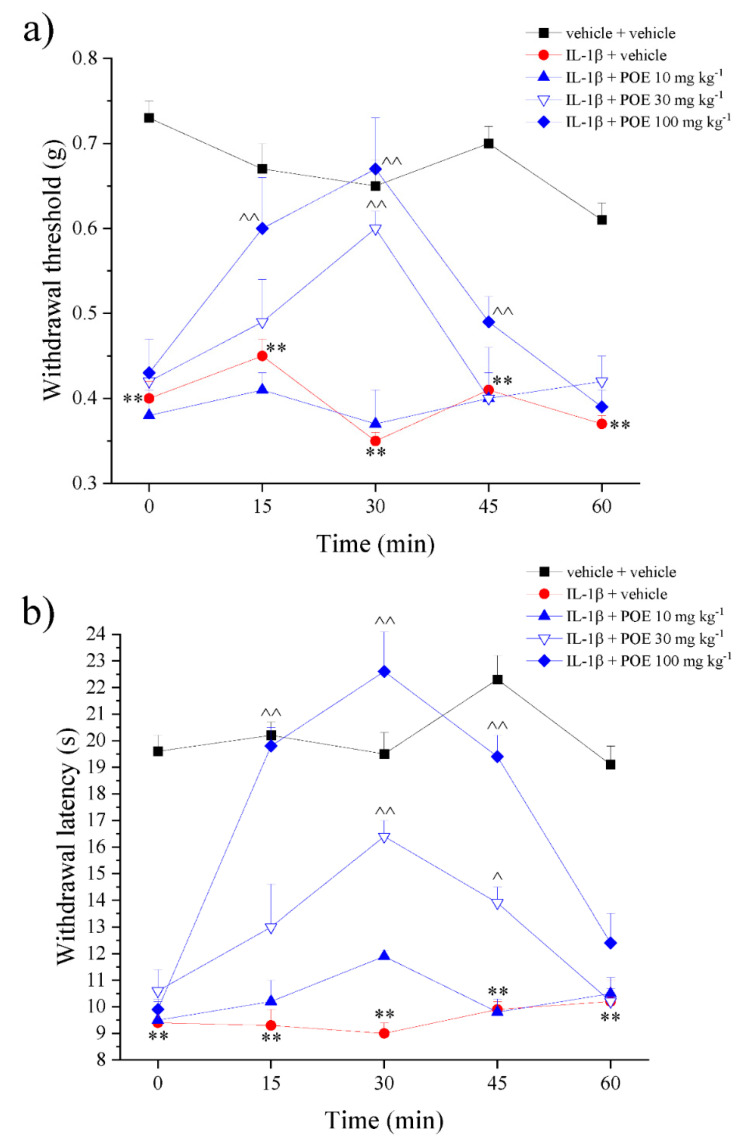
POE effects against IL-1β-induced pain. IL-1β was intraplantarly injected; 2 h later, POE was per os administered. Pain threshold was measured by (**a**) von Frey test and (**b**) paw pressure test over time. Results are reported as mean ± S.E.M. of 10 mice analyzed in 2 different experimental sessions. ** *p* < 0.01 vs. vehicle + vehicle; ^ *p* < 0.05 and ^^ *p* < 0.01 vs. IL-1β + vehicle.

Accordingly, POE (30 and 100 mg kg^−1^) was also effective in decreasing pain induced by the direct injection into the paw of IL-1β; efficacy was measured by both von Frey (Figure 3a) and paw pressure (Figure 3b) tests.

**Figure 4:**
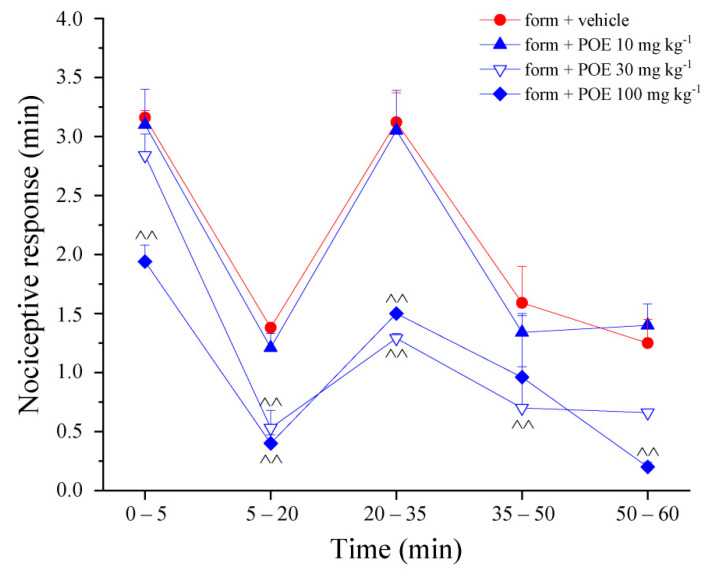
POE effects against formalin-induced pain. Formalin (form) was intraplantarly injected on time 0; in the following 60 min, the time spent lifting, favoring, licking, shaking and flinching the injected paw was recorded as nocifensive behavior. POE was p.o. administered 20 min before formalin. Control animals (vehicle + vehicle) showed 0 min as nociceptive response. Results are reported as mean ± S.E.M. of 10 mice analyzed in 2 different experimental sessions. ^^ *p* < 0.01 vs. form + vehicle.

Finally, the pain-relieving properties of POE were investigated in the formalin-induced sensitization model. Formalin shows a biphasic pain-related behavior, with an early, short-lasting first phase (0–7 min) caused by a primary afferent discharge produced by the stimulus, followed by a quiescent period and then a second, prolonged phase (15–60 min) of tonic pain related to inflammation and sensitization [17,18]. The nociceptive response was measured as the time spent in lifting, favoring, licking, shaking and flinching of the injected paw. POE 100 mg kg^−1^ was effective in both phases, the lower 30 mg kg^−1^ dose was able to significantly counteract the second prolonged phase (Figure 4).

In the writhing test, a model of visceral irritation [19] induced by the intraperitoneal injection of acetic acid able to stimulate nociceptive neurons by the release of several mediators in the peritoneal fluid [20], POE was not able to reduce the abdominal constrictions induced by the acetic acid intraperitoneal injection (Supplementary Table S1), revealing the lack of activity against irritative stimuli.

**Figure 5:**
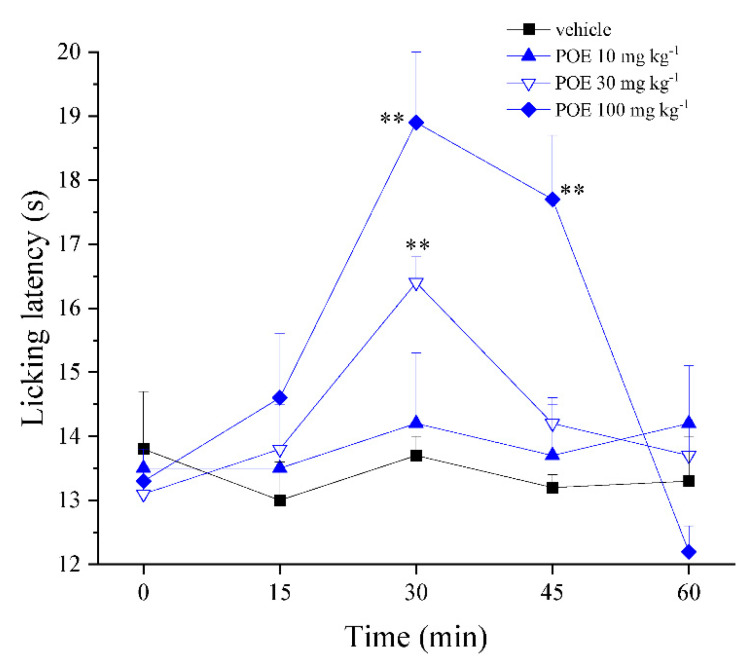
Analgesic effect of POE in naïve animals. The property to enhance the physiological pain threshold was evaluated in naïve mice by the hot-plate test. POE was p.o. administered; the time spent on a hot surface before showing nocifensive responses was recorded. Results are reported as mean ± S.E.M. of 10 mice analyzed in 2 different experimental sessions. ** *p* < 0.01 vs. vehicle + vehicle.

Based on the interesting findings collected in the hypersensitivity models mentioned above, the analgesic properties of POE were also explored in naïve animals characterized by a physiological pain threshold. Through the hot-plate test (Figure 5), it was found that POE (30 and 100 mg kg^−1^) was able to increase the physiological pain threshold evaluated as a response to a hot stimulus.

Overall, these results showed that POE had the dual characteristic of counteracting inflammation-induced hypersensitivity (hyperalgesia and allodynia) as well as enhancing the normal pain threshold by analgesic effects. To note the potency and efficacy of POE both in relieving pain and reducing paw edema is comparable to those of the widely clinically employed NSAID ibuprofen [21,22]. As discussed in a previous work as well [5], POE effectively exerts its bioactivities in the form of a phytocomplex. Thus, it is possible that its beneficial property against inflammatory pain, shown here, is due to the synergistic action of its constituents rather than that of individual bioactive compounds.

### 2.3. Effect of POE on the Inflammatory and Oxidative Mediators

The protective profile of POE was analyzed ex vivo in the paw soft tissue of carrageenan-treated animals by collecting the tissue 30 min after POE administration concurrent with the peak of pain-relieving efficacy.

The effect of POE in the tissue activity of myeloperoxidase (MPO), a primary indicator of inflammatory responses and neutrophil recruitment [23], and the tissue concentration of proinflammatory cytokines, i.e., IL-1β and TNF-α, were then evaluated. These soluble factors are able to initiate peripheral sensitization; together with reactive oxygen species (ROS) and free radicals, they activate their receptors and nociceptors terminals to decrease the pain threshold, causing hyperalgesia and inflammatory pain [24,25].

**Figure 6:**
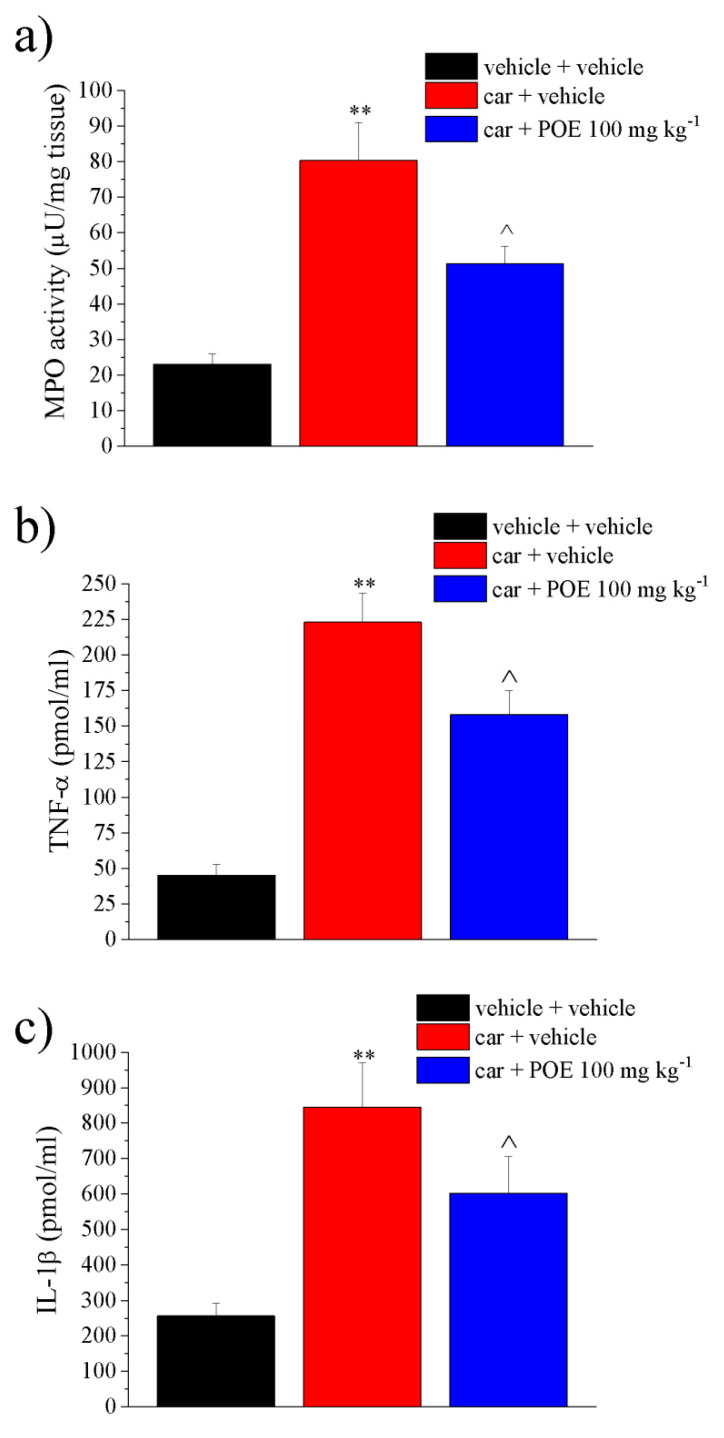
Ex vivo analysis of anti-inflammatory POE effects. Paw damage was induced by carrageenan (car, i.pl.); 2 h after car injection, POE was p.o. administered against carrageenan-induced pain and paw oedema. Two hours after the intraplantar injection of carrageenan (car), POE was p.o. administered. Thirty min later, paw tissue was collected for dosing (**a**) myeloperoxidase activity, (**b**) TNF-α and (**c**) IL-1β concentrations. Results are reported as mean ± S.E.M. of 10 mice analyzed in 2 different experimental sessions. ** *p* < 0.01 vs. vehicle + vehicle; ^ *p* < 0.05 vs. car + vehicle.

As illustrated in Figure 6a, carrageenan induced an increase in the tissue MPO activity at 80.3 ± 10.7 µU/mg compared to 23 ± 2.9 µU/mg of the vehicle; POE showed a significant inhibitory effect on the MPO activity by 50%. This result showed that POE was able to control pain in parallel with a significant decrease in tissue damage parameters.

The TNF-α concentration also increased sharply from 45.1 ± 7.8 pmol/mL of vehicle treated to 223.2 ± 20.5 pmol/mL of the carrageenan group; this increase was inhibited by 37% after POE injection (Figure 6b); similarly, POE reduced the increase in IL-1β that occurred in the control group (256.3 ± 36.1 pmol/mL) by 42% compared to the carrageenan-treated group (845.4 ± 125.1 pmol/mL), as shown in Figure 6c.

**Figure 7:**
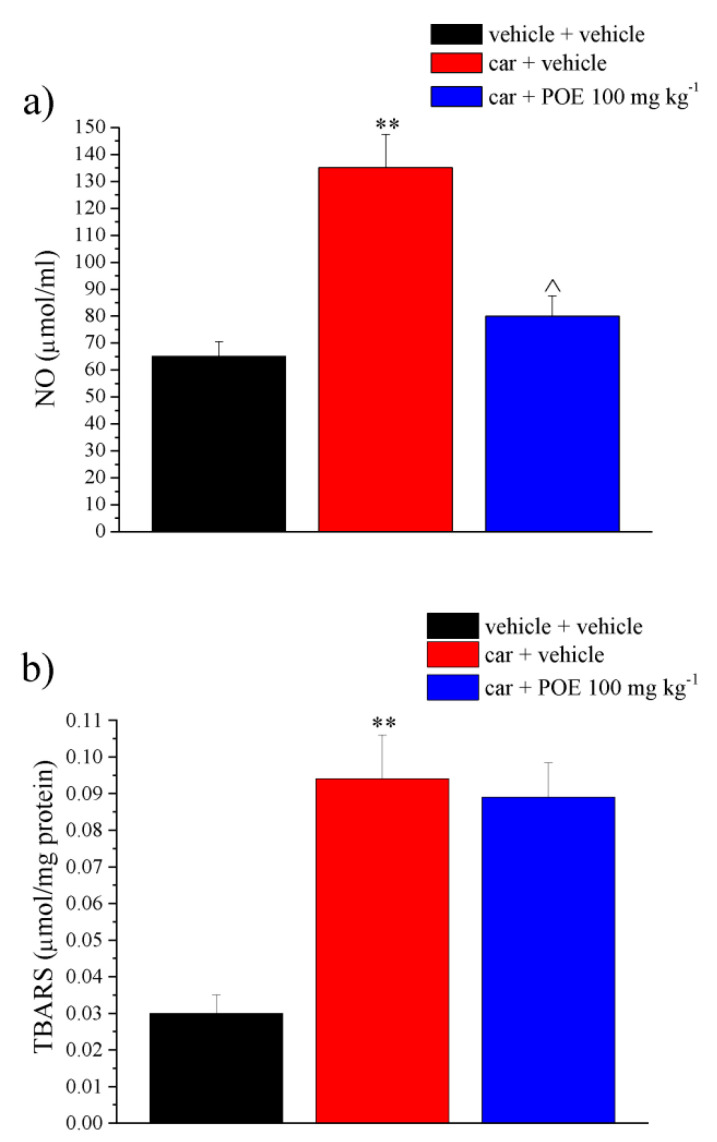
Ex vivo analysis of antioxidant POE effects. Paw damage was induced by carrageenan (car, i.pl.); 2 h after car injection, POE was p.o. administered against carrageenan-induced pain and paw oedema. Two hours after the intraplantar injection of carrageenan (car), POE was per os administered. Thirty min later, paw tissue was collected for dosing (**a**) NO levels were evaluated via nitrite and nitrate measurement according to the Griess reaction; (**b**) the peroxidation of lipids was quantified by the thiobarbituric-acid-reactive substances (TBARS) assay. Results are reported as mean ± S.E.M. of 10 mice analyzed in 2 different experimental sessions. ** *p* < 0.01 vs. vehicle + vehicle; ^ *p* < 0.05 vs. car + vehicle.

Figure 7 shows the effects of POE against carrageenan-induced redox imbalance. The inflammatory stimulus doubled the NO levels compared to the control and tripled the lipid peroxidation. As shown in Figure 7a, POE completely reduced NO levels. Contrarily, POE was found to be ineffective against lipid changes (Figure 7b).

This finding was perfectly consistent with the POE in vitro ability to suppress the expression of major inflammation-associated enzymes, including inducible nitric oxide synthase (iNOS), compromising the production of metabolites harmful to cells and tissues, such as NO, and in general the production of ROS [9].

Oxidative and nitrosative stress in tissue is a key parameter in carrageenan paw inflammation [26]. NO is a crucial mediator in the first and second phase of carrageenan-induced rat paw inflammation, which contributes to edema progression and hyperalgesia augmentation [27,28].

Macrophages and neutrophils are the potential origins of NO during inflammation, so the attenuation in the recruitment of neutrophils into the paw tissue (as assessed by myeloperoxidase activity measurements) may be responsible for POE suppression of NO increase induced by carrageenan. According to the literature [29], carrageenan also induced an increase in lipid peroxidation that could not be modified by POE probably because acute treatment was not ideal for reducing tissue damage.

## 3. Materials and Methods

### 3.1. Animals

CD-1 mice (Envigo, Varese, Italy) weighing 20–25 g at the beginning of the experimental procedure were used. Animals were housed in the Centro Stabulazione Animali da Laboratorio (University of Florence) and used at least 1 week after their arrival.

Ten mice were housed per cage (size 26 × 41 cm); animals were fed a standard laboratory diet and tap water ad libitum and kept at 23 ± 1 °C with a 12 h light/dark cycle (light at 7 a.m.).

All animal manipulations were carried out according to the Directive 2010/63/EU of the European Parliament and of the European Union Council (22 September 2010) on the protection of animals used for scientific purposes.

The ethical policy of the University of Florence complies with the Guide for the Care and Use of Laboratory Animals of the US National Institutes of Health (NIH Publication No. 85-23, revised 1996; University of Florence assurance number: A5278-01).

Formal approval to conduct the experiments described was obtained from the Italian Ministry of Health (No. 498/2017) and from the Animal Subjects Review Board of the University of Florence. Experiments involving animals have been reported according to ARRIVE guidelines [30].

All efforts were made to minimize animal suffering and to reduce the number of animals used.

### 3.2. P. oceanica Extract (POE) Preparation

The leaves of *P. oceanica* were extracted as previously described [5]. Briefly, 1 g of *P. oceanica* dried leaves were minced and suspended overnight in 10 mL of EtOH/H_2_O (70:30 *v/v*) at 37 °C under stirring and subsequently at 65 °C for 3 h.

Hydrophobic compounds were removed from the hydroalcoholic extraction by repeated shaking in n-hexane (1:1), whereas the recovered hydrophilic fraction was dispensed in 1 mL aliquots and then dried. A single batch of *P. oceanica* extract was dissolved in 0.5 mL of EtOH/H_2_O (70:30 *v/v*) before use and is hereafter referred to as POE.

Freshly dissolved POE was characterized for its total polyphenol (TP) and carbohydrate (TC) content and for its antioxidant and free-radical scavenging activities. Briefly, the Folin-Ciocalteau’s and phenol/sulfuric acid methods were used to determine the TP and TC values of POE, respectively [5,6]. Gallic acid (0.5 mg/mL) and D-glucose (1 mg/mL) were used as reference to determine TP and TC values, respectively.

The antioxidant and free-radical scavenging activities of POE were established using ferric reducing/antioxidant power assays (FRAP) and DPPH, respectively [5,6]. Ascorbic acid (0.1 mg/mL) was used as a reference to evaluate both activities.

### 3.3. POE Administration

POE extract was suspended in 1% carboxymethylcellulose sodium salt (CMC; Sigma-Aldrich, Milan, Italy) and acutely administered per os (p.o.) in a dose ranging from 10 to 100 mg kg^−1^. Control animals were treated with vehicle.

### 3.4. Carrageenan-Induced Pain and Paw Oedema in Mice

The acute inflammatory response was induced by an intraplantar injection of carrageenan (Sigma-Aldrich, Milan, Italy) in the right hind paw (car: 300 μg/80 µL, i.pl.) or vehicle (V: sterile 0.9% saline, 80 µL, i.pl.) [31]. Two hours later, POE extract was suspended in 1% carboxymethylcellulose sodium salt (CMC) and orally administered.

Pain threshold was measured before (time 0) and after (15, 30, 45 and 60 min) POE treatment. Concomitantly, to evaluate the oedema, the paw thickness was measured using a digital caliper and expressed as mm [32]. In a separate experimental setting, animals were sacrificed 30 min after POE administration, the soft tissue of the paw was collected and frozen for evaluating anti-inflammatory and antioxidant properties.

### 3.5. Formalin-Induced Pain

Mice received formalin (1.25% in saline, 30 μL) in the dorsal surface of one side of the hind paw. Each mouse, randomly assigned to one of the experimental groups, was placed in a plexiglass cage and allowed to move freely. A mirror was placed at a 45° angle under the cage to allow full view of the hind limbs. Lifting, favoring, licking, shaking and flinching of the injected paw were recorded as nocifensive behavior [33]. The total time of the nociceptive response was measured up to 60 min after formalin injection and expressed in minutes (mean ± S.E.M.). Mice received vehicle (1% CMC) or different doses of POE 20 min before formalin injection.

### 3.6. IL-1β-Induced Pain

Interleukin-1β (IL-1β) (R&D Systems Inc., Minneapolis, MN, USA) was i.pl. injected in the right hind paw (IL-1β 0.05 U/80 µL); control animals received sterile 0.9% saline, 80 µL, i.pl.) [34]. Two hours later, POE extract was suspended in 1% carboxymethylcellulose sodium salt (CMC) and orally administered. The mechanical allodynia and hyperalgesia was measured before (time 0) and after (15, 30, 45 and 60 min) POE treatment by the von Frey test and paw pressure test, respectively.

### 3.7. Von Frey Test

The animals were placed in 20 × 20 cm Plexiglas boxes equipped with a metallic meshy floor, 20 cm above the bench. A habituation of 15 min was allowed before the test. An electronic von Frey hair unit (Ugo Basile, Varese, Italy) was used: the withdrawal threshold was evaluated by applying force ranging from 0 to 5 g with a 0.2 g accuracy. Punctuate stimulus was delivered to the mid-plantar area of each anterior paw from below the meshy floor through a plastic tip and the withdrawal threshold was automatically displayed on the screen.

The paw sensitivity threshold was defined as the minimum pressure required to elicit a robust and immediate withdrawal reflex of the paw. Voluntary movements associated with locomotion were not taken as a withdrawal response. Stimuli were applied on each anterior paw with an interval of 5 s. The measure was repeated 5 times, and the final value was obtained by averaging the 5 measures [35,36].

### 3.8. Paw Pressure Test

Mechanical hyperalgesia was determined by measuring the latency in seconds to withdraw the paw away from a constant mechanical pressure exerted onto the dorsal surface [37]. A 15 g calibrated glass cylindrical rod (diameter = 10 mm) chamfered to a conical point (diameter = 3 mm) was used to exert the mechanical force. The weight was suspended vertically between two rings attached to a stand and was free to move vertically. A single measure was made per animal. A cutoff time of 40 s was used.

### 3.9. Hot-Plate Test

Analgesia was assessed using the hot plate test. With minimal animal–handler interaction, mice were taken from home-cages and placed onto the surface of the hot plate (Ugo Basile, Varese, Italy) maintained at a constant temperature of 49 °C  ±  1 °C. Ambulation was restricted by a cylindrical Plexiglas chamber (diameter, 10 cm; height, 15 cm), with open top. A timer controlled by a foot peddle began timing response latency from the moment the mouse was placed onto the hot plate. Pain-related behavior (licking of the hind paw) was observed, and the time (seconds) of the first sign was recorded. The cutoff time of the latency of paw lifting or licking was set at 40 s [38].

### 3.10. Abdominal Constriction Test

Mice were injected i.p. with a 0.6% solution of acetic acid (10 mL kg^−1^), according to Koster et al. [39]. The number of stretching movements was counted for 10 min, starting 5 min after acetic acid injection. POE was injected 20 min before acetic acid.

### 3.11. Myeloperoxidase (MPO) Activity Assay

Tissue samples were homogenized in a solution containing 0.5% hexa-decyl-trimethyl-ammonium for 1 min. After three freeze-thawing cycles, samples were sonicated for 30 s, centrifuged for 30 min at 10,000× *g*. One hundred microliter of supernatant with 2.9 mL of solution containing O-dianisidine, buffer phosphate (pH 6) and H_2_O_2_ were mixed and after 5 min, 100 mL of chloridric acid solution (1.2 M) was added. Samples’ absorbance was read spectrophotometrically at a 400 nm wavelength [40].

### 3.12. Tumor Necrosis Factor (TNF)-α and Interleukin (IL)-1β Assessment

The hind paw tissue levels of IL-1β, IL-6 and TNF-α were measured using the ELISA kits (Rat IL-1β, IL-6 and TNF-α, Biolegend, CA, USA) based on the manufacture’s guideline. In summary, the frozen hind paw tissue samples were homogenated in RIPA buffer. After centrifugation, the supernatants were incubated in the wells and after washing, diluted streptavidin-HRP-conjugated anti-rat IL-1β, IL-6 or TNF-α were added. Finally, after adding stop solution, the absorbance was read at 450 nm using an ELISA reader. The concentration of the cytokines was expressed as pg/mL of tissue.

### 3.13. Nitric Oxide (NO) Assay

NO swiftly oxidized to nitrite and nitrate subsequent to its creation. The level of total NO was evaluated via nitrite and nitrate measurement according to the Griess reaction [40]. In this colorimetric method, the final product absorbance can be determined at a wavelength of 540 nm in a microplate reader.

### 3.14. Lipid Peroxidation (Thiobarbituric Acid-Reactive Substances (TBARS) Assay)

The TBARS determination was carried out in paw tissue homogenate in PBS at the final concentration of 10% *w/v*. Then, FeCl_3_ (20 μM, Sigma-Aldrich, St. Louis, MO, USA) and ascorbic acid (100 μM, Sigma-Aldrich) were added to obtain the Fenton reaction.

At the end of incubation, the mixture was added to 4 mL reaction mixture consisting of 36 mM thiobarbituric acid (Sigma-Aldrich) solubilized in 10% CH_3_COOH, 0.2% SDS, and pH was adjusted to 4.0 with NaOH. The mixture was heated for 60 min at 100 °C, and the reaction was stopped by placing the vials in an ice bath for 10 min. After centrifugation (at 1600× *g* at 4 °C for 10 min) the absorbance of the supernatant was measured at 532 nm and 550 nm (PerkinElmer spectrometer, Milan, Italy), and TBARS were quantified in μmoL/mg of total proteins using 1,1,3,3-tetramethoxypropane as the standard [41].

### 3.15. Statistical Analysis

Behavioral measurements were performed on 10 mice for each treatment carried out in 2 different experimental sets. All assessments were made by researchers blinded to animal treatments. Results were expressed as mean ± (S.E.M.) with one-way analysis of variance. A Bonferroni’s significant difference procedure was used as a post hoc comparison. *p*-values < 0.05 or < 0.01 were considered significant. Data were analyzed using the Origin 9 software (OriginLab, Northampton, MA, USA).

## 4. Conclusions

Recent evidence has revealed that POE works as a mixture of compounds capable of synergistically evoking an effective and totally safe in vitro response for cells against inflammation.

This study represents the first attempt to provide pharmacological evidence for POE ability to relieve inflammatory pain in in vivo animal models alongside with a decrease in inflammatory and oxidative markers. In particular, POE was found to be effective in a dose-dependent manner after a single oral administration in different models of acute inflammatory pain.

Faced with the relentless demand for new alternative natural analgesic and anti-inflammatory agents, the cell-safe POE profile, described in numerous in vitro studies, together with its analgesic and anti-inflammatory properties, makes this phytocomplex an excellent candidate for continuing the investigation of the potential use of POE in the management of painful inflammatory disease in order to reduce the use of conventional drugs and, consequently, their side effects.

## Data Availability

The data presented in this study are available on request from the corresponding author.

## Supplementary Materials

The following are available online at https://www.mdpi.com/1660-3397/19/2/48/s1, Table S1: Effect of acute administration of POE on acetic-acid-induced abdominal constrictions in mice: writhing test.
