# Supplementary material for: Efficacy of Posidonia oceanica Extract against Inflammatory Pain: In Vivo Studies in Mice
Source: Mar Drugs. 2021 Jan 21;19(2):48. doi: 10.3390/md19020048 (PMC7909763; doi:10.3390/md19020048)
Supplement: Supplementary file 1 [file marinedrugs-19-00048-s001.pdf]

Supplementary material

Table S1. Mice were injected intraperitoneally (i.p.) with a 0.6 % solution of acetic acid. The number of stretching movements was counted for 10 min, starting 5 min after acetic acid injection. POE was suspended in 1% carboxymethylcellulose sodium salt (CMC) and orally administered 20 min before acetic acid injection. Measurements were performed on 10 mice for each treatments carried out in 2 different experimental sets.

Treatment	Number of stretching
Acetic ac. + vehicle	35.0 ± 1.5
Acetic ac. + POE 100 mg/kg	35.7 ± 4.8
